# Two explanations for the compliant running paradox: reduced work of bouncing viscera and increased stability in uneven terrain

**DOI:** 10.1098/rsbl.2010.0175

**Published:** 2010-03-24

**Authors:** Monica A. Daley, James R. Usherwood

**Affiliations:** Structure and Motion Laboratory, Royal Veterinary College, Hawkshead Lane, Hatfield, Hertfordshire AL9 7TA, UK

**Keywords:** locomotion, cost of transport, economy, robustness, mass-spring, leg swing

## Abstract

Economy is a central principle for understanding animal locomotion. Yet, compared with theoretical predictions concerning economy, animals run with compliant legs that are energetically costly. Here, we address this apparent paradox, highlighting two factors that predict benefits for compliant gaits: (i) minimizing cost of work associated with bouncing viscera; and (ii) leg control for robust stability in uneven terrain. We show that consideration of the effects of bouncing viscera predicts an energetic optimum for relatively compliant legs. To compare stability in uneven terrain, we introduce the normalized maximum drop (NMD), a measure based on simple kinematics, which predicts that compliant legs allow negotiation of relatively larger terrain perturbations without failure. Our model also suggests an inherent trade-off in control of leg retraction velocity (*ω*) for stability: low *ω* allows higher NMD, reducing fall risk, whereas high *ω* minimizes peak forces with terrain drops, reducing injury risk. Optimization for one of these factors explicitly limits the other; however, compliant legs relax this trade-off, allowing greater stability by both measures. Our models suggest compromises in leg control for economy and stability that might explain why animals run with compliant legs.

## 1. Introduction

Animals could move in a vast number of ways, but use only a few. Terrestrial animals use mechanically similar gaits despite differences in morphology and size (Cavagna *et al*. 1977; Heglund *et al*. 1982; Gatesy & Biewener 1991; Farley *et al*. 1993; Usherwood *et al*. 2008). Minimizing energy cost is one critical factor—animals select gaits that cost less energy to get from point A to point B (e.g. Hoyt & Taylor 1981; Bertram & Ruina 2001). Yet, current models for economy do not fully explain the movement strategies of animals.

### (a) A paradox from a theoretical perspective

**Figure 1.**
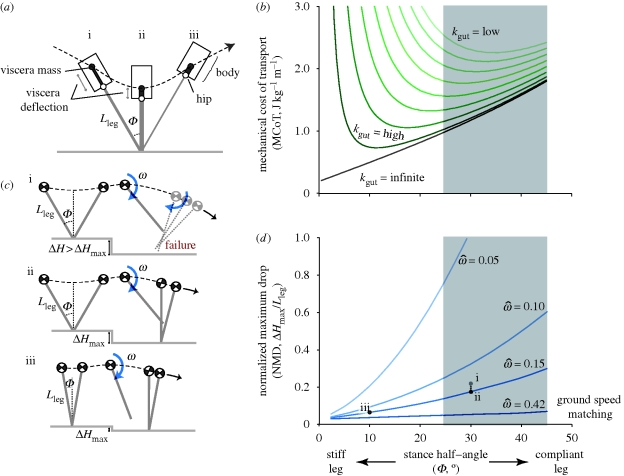
Two factors that may account for the compliant legs and large stance angles used by running animals. (*a*,*b*) A model that includes mechanical work of the legs and hysteresis losses from bouncing ‘viscera’ (*a*), suggests that compliant legs are favourable for economy. (*b*) For infinite viscera stiffness (black line), equivalent to a point mass model, mechanical cost of transport (MCoT) increases with stance half-angle (*Φ*), and impulsive running with an infinite *k*_leg_ (*Φ* = 0) is energetically optimal. If the viscera dissipate energy (green lines), however, compliant legs become favourable for economy. (*c*,*d*) Compliant legs also provide robust stability in uneven terrain. Normalized maximum drop (NMD) estimates the maximum drop relative to leg length (*Δ*H_max_/*L*_leg_) before the leg misses stance entirely ((i) in *c*,*d*). For a fixed running speed, swing period and mean leg retraction velocity (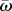, shown as dimensionless 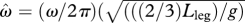) compliant legs ((ii) in *c*,*d*) have higher NMD than stiff legs ((iii) in *c*,*d*). Grey box in (*b*,*d*) indicates approximate *Φ* range used by animals (Farley *et al*. 1993).

At high enough speeds and stride lengths, a simple point mass model of bipedal locomotion suggests that an infinitely stiff, straight leg with zero sweep angle (‘impulsive running’, Srinivasan & Ruina 2006) minimizes mechanical cost of transport (MCoT) (figure 1*b*, black line), because stiff legs with small stance angles reduce fluctuations in forward velocity and kinetic energy. But animals' legs operate over a relatively compliant range that deviates from the impulsive running optimum (McMahon & Cheng 1990; Farley *et al*. 1993).

One explanation for compliant gaits is a force or stiffness limit to biological tissues, and there is evidence that peak forces limit top running speeds in humans (Weyand *et al*. 2000; Usherwood & Wilson 2006). If this were the only explanation, however, we might expect all animals to operate near a constant force or stiffness limit at all sizes and speeds. Instead, animals use different compliance strategies depending on body size and speed (Biewener 1989; Gatesy & Biewener 1991; Farley *et al*. 1993).

### (b) A paradox from a biological perspective

Locomotion is inherently more costly for small animals (per unit weight) because their short legs require that they take more steps per distance and use higher step frequencies at a given speed (Kram & Taylor 1990; Pontzer 2007). It is surprising, then, that small animals run with crouched, compliant legs (‘Groucho running’), which have lower muscle mechanical advantage and higher energy cost (McMahon *et al*. 1987; Biewener 1989; Gatesy & Biewener 1991). We might expect small animals to minimize cost by using straight, stiff legs.

Do compliant legs provide benefits that push animals away from impulsive running? Here, we highlight two factors that predict benefits for compliant gaits: (i) minimizing cost of work associated with bouncing viscera; and (ii) leg control for robust stability in uneven terrain. These factors might explain the range of leg compliance observed among animals.

## 2. Material and methods

We develop simple models to compare running with stiff versus compliant legs, based on the well-recognized mass spring model for running (e.g. McMahon & Cheng 1990; Farley *et al*. 1993). For detailed model equations, see the electronic supplementary material. We compare steady running at a fixed speed and swing duration for a range of leg stiffness (*k*_leg_), resulting in a range of stance half-angles (*Φ*, figure 1). Within species, swing duration tends to remain relatively constant across running speeds (Gatesy & Biewener 1991). Fixing swing duration in the model allows us to investigate the effects of stance leg compliance on force and work requirements of locomotion, independent from factors in swing leg control.

We also develop an analytical approximation for a mass-spring system, extended to include energy fluctuations of bouncing ‘viscera’ for a range of gut stiffness (*k*_gut_). This model considers the consequences of compliance above the hips, and so contrasts with previous models of bipedal gaits that focus on leg compliance (e.g. Alexander 1992). Here, we assume that a fraction of body mass is suspended above the hips from a dissipative spring with stiffness *k*_gut_. The parameter *k*_gut_ describes the overall compliance of all tissue mass not rigidly attached to the legs. The results presented (figure 1) are intended only as a proof of concept: the range of *k*_gut_ values used here is arbitrary; actual values for gut stiffness, hysteresis and mass await empirical evidence.

Our models assume two features of legged locomotion: (i) legs resist only compressive loads during stance (no tension or torque); and (ii) a minimum swing duration governed by a maximum leg angular velocity. We compare cost using the MCoT, the total mechanical work per unit body mass and distance travelled (figure 1*b*). The specific results shown are for human sprinting, with the following fixed parameters: body mass (*m*) = 80 kg, leg length (*L*_leg_) = 1 m, gravity (*g*) = −9.81 ms^−2^, average forward velocity = 10 ms^−1^ and leg swing period = 0.315 s; however, the general patterns hold across running speeds.

## 3. Results and discussion

### (a) A compromise between external mechanical work and the work of bouncing viscera

One possible account for compliant gaits is hysteresis losses owing to loading of viscera and any other compliant non-locomotor tissue that animals carry. We model this assuming that the viscera deflect in the direction of the leg force, dissipating energy (figure 1*a*(i)(ii)). The legs produce net positive work to restore energy dissipated by the viscera (figure 1*a*(ii)(iii)). The energy lost depends on the properties of the viscera (stiffness, hysteresis and mass), and of the legs (stiffness and sweep angle). Stiffer legs lead to higher leg forces and greater energy dissipation by the viscera, increasing MCoT (figure 1*b*, green lines). If the viscera are relatively massless, elastic or stiff, their losses are low. In this case, stiff legs are favourable. If the viscera dissipate substantial energy, compliant legs are favourable.

While stiff legs result in low leg-energy fluctuations (figure 1*b*, black line), they also require high peak leg forces. High peak leg forces cause large gut deflections and energy dissipation (electronic supplementary material, equations (4)–(14)). Consequently, for any given value of *k*_gut_, energy lost by viscera relates closely to peak leg force, and thus the inverse of contact time. The model presented here is consistent with the finding that metabolic cost relates to the timing and magnitude of ground forces (Kram & Taylor 1990; Pontzer 2007), and suggests that compliant viscera with hysteresis may be one of the primary ultimate sources for this ‘cost of force’. Unlike that of leg muscle forces, behavioural or evolutionary changes in gearing cannot ameliorate this cost; however, compliant legs would reduce the energy dissipated by viscera (figure 1*b*). The model also predicts specific selective pressures for fast and economic runners: above-hip structures should be light, stiff and as elastically supported as possible.

### (b) A compromise between external mechanical work and robust stability in uneven terrain

Another possible account for compliant gaits is improved stability in uneven terrain. Here, we consider the implications of leg compliance for robust stability. As a measure of robustness, we consider the maximum vertical terrain drop before missing a stance event (*ΔH*_max_). It may not be critical for animals to maintain a steady trajectory from stride to stride in uneven terrain, but it is reasonable to assume that they avoid falls. A sudden terrain drop can cause the leg to miss stance completely before a neural response is possible, increasing the likelihood of a catastrophic fall.

Both swing and stance dynamics influence stability. A sudden change in terrain height alters the timing of ground contact, which marks the transition from swing to stance. Recent work suggests that animals often maintain mass-spring dynamics during stance in uneven terrain (Seyfarth *et al*. 2003; Daley & Biewener 2006). This provides some intrinsic stability. Swing leg control also plays an important role. Rather than protracting the leg to the exact position for contact, animals swing the leg forward past this point and then retract it until contact. Leg retraction leads to automatic adjustment of leg contact angle with changes in terrain height (Seyfarth *et al*. 2003). Analysis of this effect leads to the prediction that animals should use high leg retraction velocities to improve stability (Seyfarth *et al*. 2003).

Yet, leg retraction also inherently limits robustness, as measured by *ΔH*_max_. With a drop in terrain, leg retraction leads to a more vertical ground contact angle (Seyfarth *et al*. 2003; Daley & Biewener 2006). A steeper contact angle reduces leg loading because the total impulse applied by the leg is roughly proportional to the angle between leg and the body velocity vector (Daley & Biewener 2006). When the angle between the leg and the body velocity is greater than 90°, the leg cannot be loaded and misses stance entirely. For high running speeds, a vertical contact angle approximates the limit for a stance event (see the electronic supplementary material).

Control of leg retraction velocity relative to leg compliance may be critical for robust stability in uneven terrain. We introduce the normalized maximum drop (NMD) as a simple kinematic measure of a runner's ability to negotiate uneven terrain (figure 1*c*,*d*). NMD may be useful for comparing robustness among animals based on simple kinematics, without complex dynamic simulations. It is an estimate of *ΔH*_max_ relative to leg length (*L*_leg_) for which the intended stance leg successfully makes any contact:
3.1
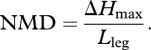

*ΔH*_max_ can be estimated from the stance half-angle (*Φ*) and the average retraction velocity as the leg approaches the ground (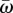). The time required for the leg to reach vertical is
3.2
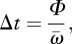

and simple ballistics dictate
3.3
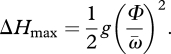

For drops larger than *ΔH*_max,_ the leg misses the intended stance phase and the body falls until the next leg makes contact (figure 1*c,d*(i)). Animals with compliant legs (figure 1*c,d*(ii)) have a higher NMD than those with stiffer legs (figure 1*c,d*(iii)). If animals use similar 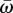, compliant gaits are favourable for robustness in uneven terrain—an intuitive outcome for anyone who has run over rough terrain at night.

Animals could use low 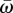 to increase NMD (reaching NMD = infinity for 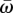 = zero), suggesting this as a possible strategy for increased robustness. However, low 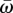 requires the leg to endure higher peak leg forces for a given drop height, which may increase injury risk. This is owing to the inherent relationship between body velocity, leg orientation and leg loading (see the electronic supplementary material). High *ω* protects the leg against high forces, but increases the likelihood of reaching the ‘no-contact’ condition defined by NMD, increasing fall risk. These findings suggest an inherent trade-off in leg retraction control: low *ω* reduces fall risk, whereas high *ω* reduces injury risk. Optimization for one of these factors inherently limits the other.

The leg retraction velocity used by animals probably reflects compromise among numerous factors in addition to stability. Leg retraction influences economy through ground speed matching, which may reduce collisional energy loss (Raibert 1986; Herr *et al*. 2002). Maximum *ω* and leg angular acceleration are probably constrained by mechanical or energetic limits (Doke *et al*. 2005). In level running, humans and pheasants use similar dimensionless leg retraction velocities 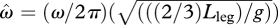, between 0.04 and 0.16 (Blum *et al*. 2010). These values appear to be more consistent with predictions for robust stability than ground speed matching (figure 1*d*). Future work should test whether animals vary *ω* depending on terrain conditions.

## 4. Conclusions

Compromises in leg control for economy and robust stability might explain why animals run with compliant legs. The optimal compliance for economy depends on viscera mass and hysteresis. The optimal compliance for robust stability probably depends on the roughness of terrain relative to body size. Our models provide a framework to develop explicit, testable predictions of optimal leg compliance depending on body size, morphology, running speed and terrain.
